# Fertility Outcome and Safety of Ethiodized Poppy Seed Oil for Hysterosalpingography in 1,053 Infertile Patients: A Real-World Study

**DOI:** 10.3389/fmed.2022.804494

**Published:** 2022-04-15

**Authors:** Hongjiang Li, Yannan Ren, Jianxiang Yan, Meiling Huang, Bolin Zheng, Xiangmin Luo, Suzhen Huang, Siqing Cai

**Affiliations:** Department of Radiology, The Second Affiliated Hospital of Fujian Medical University, Quanzhou, China

**Keywords:** ethiodized poppy seed oil, hysterosalpingography, infertile patients, pregnancy rate, livebirth rate

## Abstract

**Objective:**

Ethiodized poppy seed oil for hysterosalpingography (HSG) is reported to display some therapeutic effect on infertility, but big a sample-size study under real clinical settings is still lacking to verify the speculation. Thus, this real-world study enrolled 1,053 infertile patients who underwent ethiodized poppy seed oil-based HSG to explore its fertility enhancement value.

**Method:**

A total of 1,053 infertile patients who underwent HSG using ethiodized poppy seed oil as the contrast medium were retrospectively analyzed. The live birth rate and 3-, 6-, 12-month and total pregnancy rate were retrieved. Besides, adverse events during and after HSG were recorded.

**Results:**

The 3-, 6-, 12-month and total pregnancy rate was 22, 36.8, 50, and 53.8%, respectively. The total live birth rate was 42.7%. Sub-group analyses showed that pregnancy rate was 53.7, 53.8, 54.1, and 62.4% in subgroups of primary infertility patients, secondary infertility patients, infertility patients with fallopian tube disease, and infertility patients with unknown cause, respectively. Meanwhile the live birth rate was 44.3, 41.3, 41.5, and 59.2% in these subgroups, separately. Multivariate logistic regression analysis disclosed that BMI ≥ 24 kg/m^2^, history of dysmenorrhea, and abnormity of sperm count or motility-related infertility were independently correlated with reduced pregnancy rate and livebirth rate (All *P*s < 0.05). Adverse events mainly included pain (20.6%) and interstitial reflux (7.9%), which were mild and tolerable.

**Conclusion:**

Ethiodized poppy seed oil for HSG discloses a satisfying fertility outcome with a tolerable safety profile in infertile patients; meanwhile, this effect might be influenced by BMI, history of dysmenorrhea, and paternal abnormity of sperm.

## Introduction

Infertility, defined as unsuccessful pregnancy after 1 year of unprotected sexual intercourse, presents with high prevalence and affects 10–15% couples worldwide; while in China, 12.5–15.5% couples suffered from infertility with an upward trend incidence during the past 2 decades (1–5). The pathogenesis of infertility includes many aspects, among which, fallopian tube diseases, including tubal obstruction and abnormality, are one of the most common reasons for infertility, and account for 25–43.7% of infertility cases (6, 7). Hysterosalpingography (HSG) is known as a precise tool for diagnosis of tubal patency; however, interestingly, apart from its application in diagnosis of tubal diseases, recent studies have also reported its therapeutic effect in infertile patients to some extent (3, 8, 9).

Moreover, the fertility enhancement effect after HSG is diverse according to the contrast agent used for HSG process. In details, the application of an oil-based contrast agent always indicates higher pregnancy rate than that of a water-based contrast agent (3, 9). For instance, in a small sample-size study, HSG with lipiodol brings about high overall pregnancy rate and live birth pregnancy rate among couples with unexplained and endometriosis-related infertility (10). Besides, a multicenter, randomized controlled study also shows that the application of ethiodized poppy seed oil enhances ongoing pregnancy rate and live birth rate compared with the application of meglumine ioxitalamic acid during HSG (3). Unfortunately, most relevant studies either small have a sample size or are randomized controlled trials, while big sample-size studies under real clinical circumstances to verify the fertility enhancement effect of ethiodized poppy seed oil for HSG are still lacking.

Thus, this real-world study analyzed 1,053 infertile patients who underwent HSG using ethiodized poppy seed oil as the contrast agent, and aimed to assess the effect of ethiodized poppy seed oil for HSG on fertility as well as its safety in infertile patients.

## Methods

### Subjects

This study retrospectively reviewed 1,053 infertile patients who underwent HSG in a hospital from March 2019 to October 2019. The screening criteria were as follows: (a) women who had sex regularly over a year without using any form of contraception but were still not pregnant; (b) aged between 21 and 45 years old; (c) received HSG with ethiodized poppy seed oil; (d) with at least one fallopian tube open. Patients were excluded from the study if they had massive missing data on clinical characteristics, efficacy, and safety assessment. This study was approved by an institutional review board, and all the patients signed informed consent.

### HSG Procedure

Ethiodized poppy seed oil was used for HSG in the study. HSG was performed within 3–7 days after complete cessation of menstruation. The patients were required to meet the basic conditions of nonsexual intercourse after menstruation and cleanliness of grades I-II in routine preoperative leucorrhea examination; the mycoplasma and chlamydia were also examined if necessary. After pretreatment for prevention of fallopian tube spasm and emptying of bladder, the patients were required to maintain the lithotomy position; then, the vagina was dilated with a dilator to expose the cervix. A double-lumen balloon catheter was slowly placed into the cervix, and 2–3 ml of gases were injected into the balloon to fix the catheter. A pelvic plain film was first captured as the reference film, then the contrast agent (ethiodized poppy seed oil) was slowly injected into the catheter. During the injection, speed and pressure were appropriately adjusted according to the tolerance of the patients. When the patients began to feel distension of the lower abdomen, the second X-ray film was captured. If the development of uterine cavity or fallopian tube was ineligible, the position of the catheter was adjusted, and another 3–5 ml of the contrast agent was injected; then, the third X-ray film was taken. The fourth pelvic plain film in the decubitus position was taken 24 h after the HSG, and diffusion of the contrast medium in the pelvic cavity was observed. If the patients suffered from venous or lymphatic reflux during the HSG, the injection was stopped immediately, and the patients were ordered to rest in the decubitus position. Meanwhile, analgesia and anti-allergy treatments were given if necessary until symptoms were relieved. After discharged, the patients were advised to abstain from both the sexual life and the bath within 2 weeks. Also, they were advised to wait for natural pregnancy without other treatments within 3 months after HSG. After the films were taken, the images were evaluated by chief physicians for diagnosis workup.

### Collection of Clinical Data

According to medical documents, clinical data were collected for analysis, including demographic characteristics (age and body mass index, BMI), history of dysmenorrhea, reproductive history (times of pregnancy and times of delivery), infertility characteristics (type of infertility and etiology of infertility), and subsequent therapy after HSG.

### Statistics Analysis

The SPSS V.19.0 software (IBM Corp., Armonk, NY, United States) and GraphPad Prism 7.02 (GraphPad Software Inc., San Diego, CA, United States) were used for statistical analyses and graph-making. Variables were described with mean ± standard deviation (SD) and number with percentage (No. %). Comparisons were checked by chi-square test and Fisher's exact test. Logistic regression analyses for pregnancy and live birth were performed, and the forward stepwise methodology was applied for screening independent prediction factors. Statistical significance was concluded if a *P* < 0.05 was presented in the corresponding analysis.

## Results

### Clinical Characteristics

The mean age of the enrolled patients was 30.3 ± 4.3 years. Besides, mean BMI was 21.8 ± 3 kg/m^2^. With respect to reproductive history, 471 (44.7%) of the infertile patients had never been pregnant, while 483 (45.9%), 88 (8.4%), and 11 (1%) of the infertile patients had 1, 2, and 3 times of pregnancy, respectively. Furthermore, 653 (62.0%), 382 (36.3%), and 18 (1.7%) of the infertile patients had 0, 1, and 2 times of delivery, respectively. With regard to the type and etiology of infertility, 479 (45.5%) of the infertile patients have primary infertility, while 574 (54.5%) of them have secondary infertility. Besides, the etiology of most of the infertile patients [498 (47.3%)] was unexplained. Meanwhile, fallopian tube disease accounted for 21.7% of the etiology of infertility. Other detailed clinical characteristics are shown in Table 1. Besides, subsequent therapy for infertile patients after HSG is listed in Supplementary Table 1.

**Table 1:** Clinical characteristics.

**Items**	**Patients (*N* = 1,053)**
**Demographic characteristics**	
Age (years), mean ± SD	30.3 ± 4.3
BMI (kg/m^2^)	
Mean ± SD (*n* = 1,035)	21.8 ± 3.0
Unknown, No. (%)	18 (1.7)
**History of dysmenorrhea, No. (%)**	
No	859 (81.6)
Yes	178 (16.9)
Unknown	16 (1.5)
**Reproductive history**	
Times of pregnancy, No. (%)	
0	471 (44.7)
1	483 (45.9)
2	88 (8.4)
3	11 (1.0)
Times of delivery, No. (%)	
0	653 (62.0)
1	382 (36.3)
2	18 (1.7)
**Infertility characteristics**	
Type of infertility, No. (%)	
Primary infertility	479 (45.5)
Secondary infertility	574 (54.5)
Etiology of infertility, No. (%)	
Immune disease	10 (0.9)
Endocrine disease	152 (14.4)
Uterine disease	150 (14.2)
Fallopian tube disease	229 (21.7)
Ovarian disease	142 (13.5)
Abnormity of sperm count or motility	145 (13.8)
Unknown cause	498 (47.3)

*SD, standard deviation; BMI, body mass index; HSG, hysterosalpingography*.

### Pregnancy and Live Birth Rate After HSG

**Figure 1:**
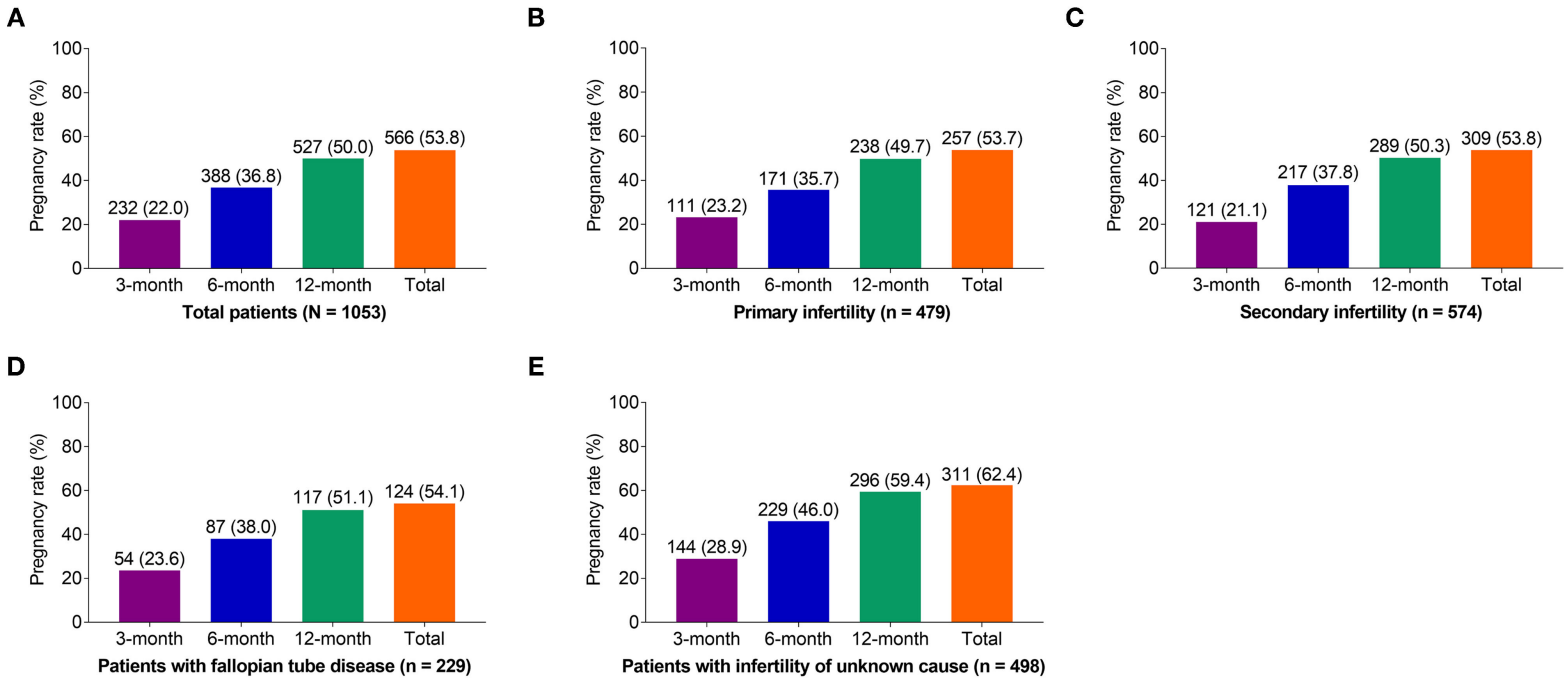
Effect of ethiodized poppy seed oil for HSG on pregnancy rate in infertile patients. Three-month, 6-month, 12-month, and total pregnancy rate after initiation of HSG in **(A)** all of the patients, **(B)** patients with primary infertility, **(C)** patients with secondary infertility, **(D)** patients with fallopian tube disease, and **(E)** patients with infertility of unknown cause. HSG, hysterosalpingography.

In general, the 3-, 6-, 12-month, and total pregnancy rates were 22, 36.8, 50, and 53.8% in all of the infertile patients (*N* = 1,053; Figure 1A). Furthermore, a subgroups analysis was conducted according to the type of infertility (primary and secondary infertility) and etiology of infertility (unknown cause and fallopian tube disease). The data disclosed that pregnancy rate was 53.7, 53.8, 54.1, and 62.4% in the primary infertility patients (*n* = 479, Figure 1B), secondary infertility patients (*n* = 574, Figure 1C), infertility patients with fallopian tube disease (*n* = 229, Figure 1D) and infertility patients with unknown cause (*n* = 498, Figure 1E), respectively.

**Figure 2:**
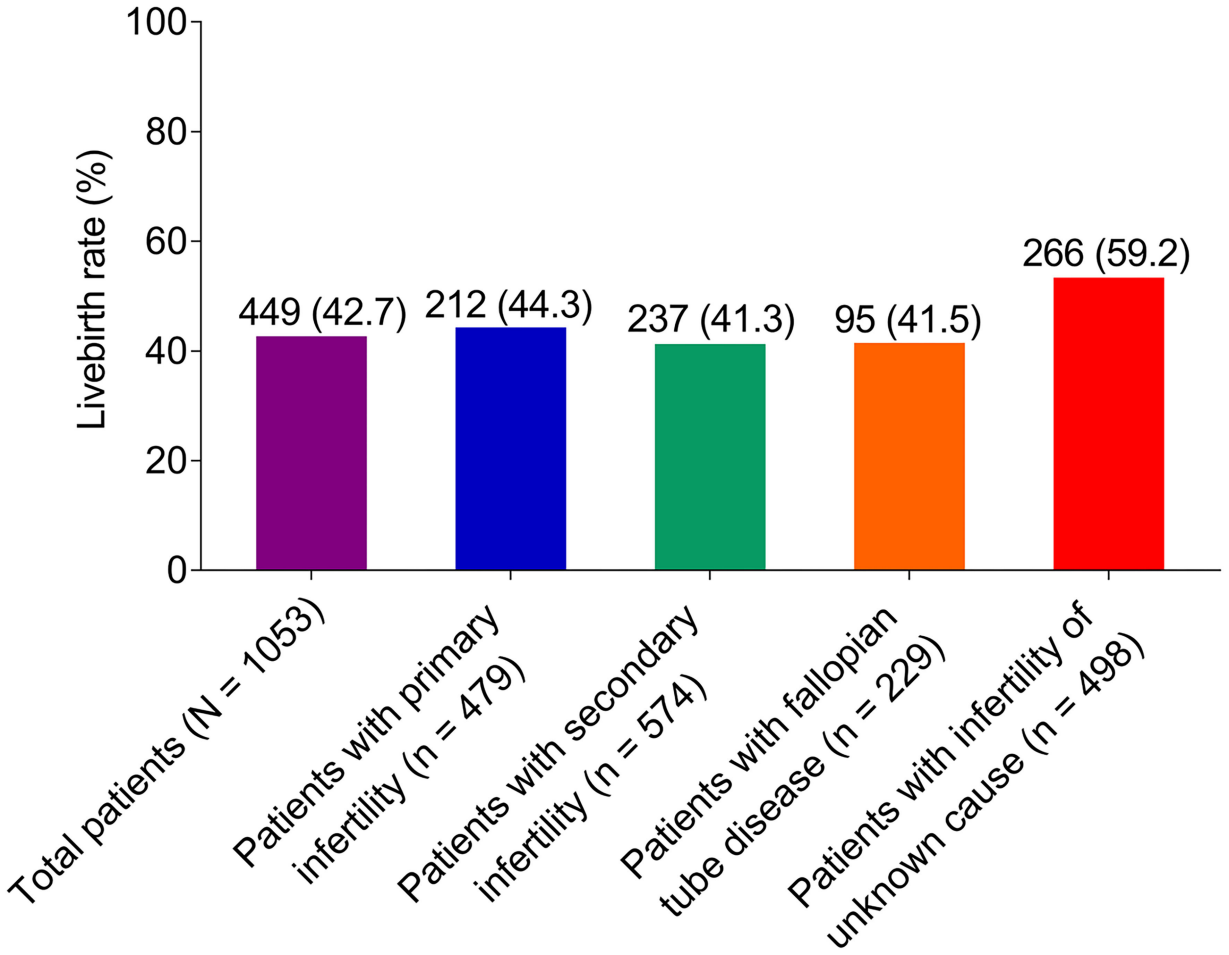
Effect of ethiodized poppy seed oil for HSG on live birth rate in infertile patients. HSG, hysterosalpingography.

With respect to live birth rate, it was 42.7% in all of the patients. Results of the subgroup analysis showed that livebirth rate was 44.3, 41.3, 41.5, and 59.2% in patients with primary infertility, patients with secondary infertility, infertile patients with fallopian tube disease, and infertile patients with unknown cause, respectively (Figure 2).

### Correlation of Clinical Characteristics With Pregnancy Rate

Body mass index (BMI) ≥ 24 kg/m^2^ (odds ratio, OR:0.633, *P* = 0.004, Table 2), history of dysmenorrhea (OR:0.561, *P* < 0.001), endocrine disease-related infertility (OR:0.560, *P* = 0.001), ovarian disease-related infertility (OR:0.605, *P* = 0.006), and abnormity of sperm count or motility-related infertility (OR:0.347, *P* < 0.001) were correlated with decline in pregnancy rate, while unknown cause of infertility was associated with elevated pregnancy rate (OR: 1.957, *P* < 0.001).

**Table 2:** Pregnancy rate among patients with different clinical characteristics.

**Items**	**Non-pregnancy**	**Pregnancy**	***P*-value**	**Crude OR (95%CI)**
**Demographic characteristics**				
Age, No. (%)			0.132	0.830 (0.651–1.058)
<30.0 years	220 (43.8)	282 (56.2)		
≥30.0 years	267 (48.5)	284 (51.5)		
BMI, No. (%)			**0.004**	0.633 (0.465–0.862)
<24.0 kg/m^2^	375 (44.1)	476 (55.9)		
≥24.0 kg/m^2^	112 (55.4)	90 (44.6)		
**History of dysmenorrhea, No. (%)**			**<0.001**	0.561 (0.405–0.778)
No	369 (43.0)	490 (57.0)		
Yes	102 (57.3)	76 (42.7)		
**Reproductive history**				
Times of pregnancy, No. (%)			0.358	1.088 (0.909–1.303)
0	217 (46.1)	254 (53.9)		
1	233 (48.2)	250 (51.8)		
2	34 (38.6)	54 (61.4)		
3	3 (27.3)	8 (72.7)		
Times of delivery, No. (%)			0.364	0.898 (0.713–1.132)
0	295 (45.2)	358 (54.8)		
1	183 (47.9)	199 (52.1)		
2	9 (50.0)	9 (50.0)		
**Infertility characteristics**				
Type of infertility, No. (%)			0.954	1.007 (0.790–1.285)
Primary infertility	222 (46.3)	257 (53.7)		
Secondary infertility	265 (46.2)	309 (53.8)		
Etiology of infertility, No. (%)				
Immune disease	8 (80.0)	2 (20.0)	0.051	0.212 (0.045–1.005)
Endocrine disease	89 (58.6)	63 (41.4)	**0.001**	0.560 (0.395–0.794)
Uterine disease	80 (53.3)	70 (46.7)	0.060	0.718 (0.508–1.015)
Fallopian tube disease	105 (45.9)	124 (54.1)	0.892	1.021 (0.761–1.369)
Ovarian disease	81 (57.0)	61 (43.0)	**0.006**	0.605 (0.424–0.865)
Abnormity of sperm count or motility	99 (68.3)	46 (31.7)	**<0.001**	0.347 (0.239–0.504)
Unknown cause	187 (37.6)	311 (62.4)	**<0.001**	1.957 (1.529–2.504)

*BMI, body mass index; HSG, hysterosalpingography. The bold values mean the statistical significance*.

In order to further determine the independent factors influencing pregnancy rate, a multivariate logistic regression analysis was performed (Table 3), which showed that BMI ≥ 24 kg/m^2^ (OR:0.619, *P* = 0.003), history of dysmenorrhea (OR:0.617, *P* = 0.005), and abnormity of sperm count or motilityrelated infertility (OR:0.436, *P* < 0.001) were independently related to reduced pregnancy rate, while unknown cause of infertility was an independent factor for increased pregnancy rate (OR: 1.469, *P* = 0.007).

**Table 3:** Multivariate logistic regression analysis for pregnancy.

**Items**	***P* value**	**Adjusted OR**	**95%CI**	
			**Lower**	**Upper**
BMI ≥24.0 kg/m^2^	0.003	0.619	0.450	0.852
History of dysmenorrhea	0.005	0.617	0.440	0.865
Etiology of infertility				
Abnormity of sperm count or motility	<0.001	0.436	0.290	0.654
Unknown cause	0.007	1.469	1.110	1.945

*BMI, body mass index; HSG, hysterosalpingography*.

Furthermore, a subgroups analysis on the association of clinical characteristics with pregnancy rate at different time points was also conducted, and the detailed findings are listed in Supplementary Table 2.

### Correlation of Clinical Characteristics With Live Birth Rate

The correlation of clinical characteristics with live birth rate was also determined, and disclosed that the following factors, namely, BMI ≥ 24 kg/m^2^ (OR:0.618, *P* = 0.004, Table 4), history of dysmenorrhea (OR:0.551, *P*= 0.001), immune disease-related infertility (OR:0.151, *P* = 0.047), endocrine disease-related infertility (OR:0.47, *P* < 0.001), uterine disease-related infertility (OR:0.602, *P* = 0.008), ovarian disease-related infertility (OR:0.449, *P* < 0.001), and abnormity of sperm count or motility-related infertility (OR:0.292, *P* < 0.001) were associated with decreased live birth rate, while unknown cause of infertility was linked to increased live birth rate (OR: 2.365, *P* < 0.001).

**Table 4:** Live birth rate among patients with different clinical characteristics.

**Items**	**Non-livebirth**	**Livebirth**	***P*-value**	**Crude OR (95%CI)**
**Demographic characteristics**				
Age, No. (%)			0.342	0.886 (0.690–1.138)
<30.0 years	250 (53.4)	218 (46.6)		
≥30.0 years	299 (56.4)	231 (43.6)		
BMI, No. (%)			**0.004**	0.618 (0.446–0.857)
<24.0 kg/m^2^	426 (52.8)	381 (47.2)		
≥24.0 kg/m^2^	123 (64.4)	68 (35.6)		
**History of dysmenorrhea, No. (%)**			**0.001**	0.551 (0.390–0.779)
No	420 (51.8)	391 (48.2)		
Yes	113 (66.1)	58 (33.9)		
**Reproductive history**				
Times of pregnancy, No. (%)			0.576	0.949 (0.788–1.141)
0	234 (52.8)	209 (47.2)		
1	266 (57.7)	195 (42.3)		
2	44 (53.0)	39 (47.0)		
3	5 (45.5)	6 (54.5)		
Times of delivery, No. (%)			0.060	0.795 (0.626–1.010)
0	325 (52.7)	292 (47.3)		
1	213 (58.7)	150 (41.3)		
2	11 (61.1)	7 (38.9)		
**Infertility characteristics**				
Type of infertility, No. (%)			0.245	0.862 (0.671–1.107)
Primary infertility	239 (53.0)	212 (47.0)		
Secondary infertility	310 (56.7)	237 (43.3)		
Etiology of infertility, No. (%)				
Immune disease	8 (88.9)	1 (11.1)	**0.047**	0.151 (0.019–1.211)
Endocrine disease	101 (70.1)	43 (29.9)	**<0.001**	0.470 (0.321–0.688)
Uterine disease	91 (65.5)	48 (34.5)	**0.008**	0.602 (0.414–0.876)
Fallopian tube disease	126 (57.0)	95 (43.0)	0.497	0.901 (0.666–1.218)
Ovarian disease	96 (71.1)	39 (28.9)	**<0.001**	0.449 (0.302–0.667)
Abnormity of sperm count or motility	108 (78.3)	30 (21.7)	**<0.001**	0.292 (0.191–0.448)
Unknown cause	209 (44.0)	266 (56.0)	**<0.001**	2.365 (1.832–3.052)

*BMI, body mass index; HSG, hysterosalpingography. The bold values mean the statistical significance*.

A multivariate logistic regression analysis was performed to determine independent factors affecting live birth rate, and showed that BMI ≥ 24 kg/m^2^ (OR:0.596, *P* = 0.002, Table 5), history of dysmenorrhea (OR:0.633, *P* = 0.01) and abnormity of sperm count or motility-related infertility (OR:0.405, *P* < 0.001) were independent factors for decreased live birth rate, and that unknown cause of infertility was independently correlated with elevated live birth rate (OR: 1.433, *P* = 0.015).

**Table 5:** Multivariate logistic regression analysis for live birth.

**Items**	***P* value**	**Adjusted OR**	**95%CI**	
			**Lower**	**Upper**
BMI ≥24.0 kg/m^2^	0.002	0.596	0.427	0.831
History of dysmenorrhea	0.010	0.633	0.446	0.898
Etiology of infertility				
Abnormity of sperm count or motility	<0.001	0.405	0.263	0.623
Unknown cause	0.015	1.433	1.072	1.917

*BMI, body mass index; HSG, hysterosalpingography*.

### Adverse Events

During and after the HSG process, the most common adverse events were pain [217 (20.6%), Table 6] and interstitial reflux [83 (7.9%)]. Meanwhile, most of them were mild with low incidence. In detail, most of the pain was mild [155 (14.7%)], while only 52 (5%) and 10 (0.9%) of the infertile patients suffered from moderate or severe pain.

**Table 6:** Adverse events.

**Items**	**Infertility patients (*N* = 1,053)**
Interstitial reflux, No. (%)	83 (7.9)
Pain, No. (%)	217 (20.6)
Pain degree, No. (%)	
No	836 (79.4)
Mild	155 (14.7)
Moderate	52 (5.0)
Severe	10 (0.9)

## Discussion

A contrast medium (so far, most frequently used contrast media include the oil-based contrast medium and the water-based contrast medium) is needed to be injected into the uterus for enhanced display during the HSG procedure. Some studies have already compared the fertility enhancement effect of HSG between the oil-based contrast medium and the water-based contrast medium in infertile patients (3, 8). However, the fertility enhancement effect of HSG using the oil-based contrast medium under real-world conditions is still not clear; therefore, this study is performed.

Hysterosalpingography (HSG), a manner by injecting the contrast medium into the uterus and fallopian tube under the X ray for observing its morphology, is widely used in diagnosis of tubal disease such as fallopian tube obstruction and fallopian tube umbrella end adhesion etc. (11, 12). Apart from its precious value in the diagnosis of uterus and fallopian tube diseases, studies also emphasize on its therapeutic effect on patients with infertility as early as 1960s (13). In the past six decades, HSG has been widely applied in treatment of infertility; interestingly, the therapeutic effect is diverse according to the contrast agent used for HSG process. To be specific, an oil-based contrast for HSG discloses better pregnant outcomes than a water-based regimen (3, 8, 9). For instance, a multicenter, randomized trial conducted in the Netherlands reveals that using an oil-based contrast for HSG achieves higher ongoing pregnancy rate (39.7 vs. 29.1%) and live birth rate (39.7 vs. 28.1%) than using a water-based contrast during HSG process (3). Another study comparing long-term pregnancy outcomes between oil-based and water-based contrasts for HSG in infertile patients shows that oil group has higher ongoing pregnancy rate (80 vs. 75%), live birth rate (74.8 vs. 67.3%), natural conception rate (41.1 vs. 34.7%), and shorter time to ongoing pregnancy (10 vs. 13.7 months) than the water group (9). Besides, a meta-analysis also shows that oil-based contrasts are associated with higher rates of ongoing pregnancy compared with water-based contrasts (8). Unfortunately, these previous studies either have a small sample size or are randomized controlled trials, but real-clinical setting studies with a large sample size to verify the fertility enhancement effect of ethiodized poppy seed oil for HSG are demanded. Thus, we conducted this real-world study, which analyzed 1,053 infertile patients who received ethiodized poppy seed oil for HSG and determined the pregnant outcomes in these patients, which showed that the total pregnancy rate and live birth rate were 53.8 and 42.7%, respectively. The possible reasons for the enhancement of fertility by the ethiodized poppy seed oil for HSG might be that: 1) the increment of Th1 cell proportion was harmful for placental implantation, while Th2 might overrule Th1 immunity and further protected a fetus and accommodated fetal and placental development by balancing Th1 immunity. A study had reported that ethiodized poppy seed oil might create a favorable environment for placental implantation and pregnancy, although regulating the proportion of Th1/Th2 cells, and thus further elevating pregnancy rate and live birth rate (14, 15); 2) the ethiodized poppy seed oil contrast medium might promote fertilization as well as placental implantation although (a) altering membrane electronegativity and micro-viscosity, which further inhibited macrophage phagocytosis and adherence; (b) reducing the maternal rejection reaction to the sperm, although decreasing the amount of dendritic cells, thus improving pregnancy outcomes (16, 17); 3) the ethiodized poppy seed oil contrast medium, during the HSG process, had a mechanical flushing effect, which might sweep the impaired fallopian tube tissue or mucous plug, thus helping the ovum reach the uterus more smoothly (3). Hence, the ethiodized poppy seed oil contrast for HSG showed a fertility-enhancement effect.

Previous studies have shown that higher BMI, history of dysmenorrhea, and abnormity of sperm count or motility-related infertility are correlated with poor pregnant outcomes (18–22). In line with the previous studies, our study also found that BMI ≥ 24 kg/m^2^, history of dysmenorrheal, and abnormity of sperm count or motility-related infertility were independently associated with declined pregnancy rate and live birth rate. These phenomena might explain that: 1) these infertile patients with higher BMI presented more frequent ovulatory dysfunction, which caused a worse pregnancy outcome (18); 2) the BMI of the infertile patients was positively associated with incidence of sexual dysfunction, thus causing lower pregnancy rate and live birth rate (23); 3) The endometriosis was more frequently observed in patients with history of dysmenorrhea compared with those without history of dysmenorrhea; meanwhile, the presence of dysmenorrhea was correlated with late-stage endometriosis, which might cause poor ability of conception (19, 24); 4) These infertile patients whose couples were complicated with abnormity of sperm count or motility were hard to form a zygote in the uterus after a normal sexual intercourse, thus causing decreased fertility (20, 21).

In an extensive study, adverse events during and after HSG include pain, interstitial reflux, nausea, emesis, colporrhagia, and so on; among which pain is the most frequent adverse event (25, 26). For instance, one study shows that after HSG, about half of patients suffered from pain, followed by colporrhagia, nausea, and interstitial reflux. In this study we also disclosed a similar discovery with a previous study. In detail, pain was the most common adverse event after the HSG process, with an incidence of 20.6%, followed by interstitial reflux with an incidence of 7.9%. Meanwhile, most of the pain was mild. The mechanism of the occurrence of pain after the HSG process might include the following aspects: 1) due to fact that HSG was an invasive surgery, apparatuses, such as catheter, might cause mechanical trauma during HSG, thus causing the occurrence of pain; 2) women might be nervous, with their muscles tightened during HSG, which might cause the HSG process to become more difficult to their surgeons, thus further forcing surgeons to exert more strength during the HSG process, hence causing higher pain intensity (25, 26). In general, using the ethiodized poppy seed oil contrast for HSG was safe and well-tolerated.

There were some limitations in this study: 1) we observed some independent factors for predicting pregnancy rate and live birth rate after ethiodized poppy seed oil-based HSG, and these findings are needed to be verified in a future validation cohort; 2) The follow-up period of this study was relatively short, and a further long-term follow-up study is needed; 3) Selection bias might exist because of regional restriction; 4) The comparison of laparoscopic tubal test with HSG using the oil-based contrast medium in the diagnostic effect of tubal disease in infertile patients should be determined in further real-world studies. 5) A control group (such as that of infertile patients receiving hysteroscopic hydrotubation or HSG using a water-based contrast medium) lacked in this study. Therefore, a further real-world study comparing the fertility enhancement effect between these two groups (HSG using an oil-based contrast medium vs. hysteroscopic hydrotubation or HSG using a water-based contrast medium) is needed.

## Conclusion

To be conclusive, ethiodized poppy seed oil for HSG discloses a satisfying fertility outcome with a tolerable safety profile in infertile patients. Meanwhile, this effect might be influenced by BMI, history of dysmenorrhea, and paternal abnormity of sperm.

## Data Availability Statement

The original contributions presented in the study are included in the article/Supplementary Material, further inquiries can be directed to the corresponding author.

## Ethics Statement

This study was approved by the Institutional Review Board of the Second Affiliated Hospital of Fujian Medical University, and all patients signed the informed consents. The patients/participants provided their written informed consent to participate in this study.

## Author Contributions

Conception and design were performed and administrative support was provided by HL and SC. Provision of study materials or patients was made by HL, YR, JY, MH, BZ, XL, SH, and SC. Collection and assembly of data were performed by HL, YR, and JY. Data analysis and interpretation were conducted by HL, YR, MH, and SC. All authors manuscript writing and final approval of the manuscript.

## Conflict of Interest

The authors declare that the research was conducted in the absence of any commercial or financial relationships that could be construed as a potential conflict of interest.

## Publisher's Note

All claims expressed in this article are solely those of the authors and do not necessarily represent those of their affiliated organizations, or those of the publisher, the editors and the reviewers. Any product that may be evaluated in this article, or claim that may be made by its manufacturer, is not guaranteed or endorsed by the publisher.
