# Supplementary material for: Fertility Outcome and Safety of Ethiodized Poppy Seed Oil for Hysterosalpingography in 1,053 Infertile Patients: A Real-World Study
Source: Front Med (Lausanne). 2022 Apr 15;9:804494. doi: 10.3389/fmed.2022.804494 (PMC9051392; doi:10.3389/fmed.2022.804494)
Supplement: Supplementary file 1 [file Table_1.DOCX]

**Supplementary table 1.** Subsequent therapy after HSG

| Items | Patients (N = 1053) |
| --- | --- |
| Subsequent therapy after HSG, No. (%) | 377 (35.8) |
| Ovulation induction | 180 (17.1) |
| Hysteroscope | 90 (8.5) |
| Artificial insemination | 40 (3.8) |
| Western medicine | 33 (3.1) |
| Vitro fertilization | 32 (3.0) |
| Chinese medicine | 29 (2.8) |
| Tubal dredge operation | 27 (2.6) |
| Peritoneoscope | 5 (0.5) |
| Selective salpingography | 2 (0.2) |
| Hydrotubation | 2 (0.2) |

HSG, hysterosalpingography.
