# Supplementary material for: Fertility Outcome and Safety of Ethiodized Poppy Seed Oil for Hysterosalpingography in 1,053 Infertile Patients: A Real-World Study
Source: Front Med (Lausanne). 2022 Apr 15;9:804494. doi: 10.3389/fmed.2022.804494 (PMC9051392; doi:10.3389/fmed.2022.804494)
Supplement: Supplementary file 2 [file Table_2.DOCX]

**Supplementary table 2.** Subgroup analysis for pregnant rate in different time

| Items | 3-month | | | 6-month | | | 12-month | | |
| --- | --- | --- | --- | --- | --- | --- | --- | --- | --- |
|  | No | Yes | *P* value | No | Yes | *P* value | No | Yes | *P* value |
| **Demographic characteristics** |  |  |  |  |  |  |  |  |  |
| Age, No. (%) |  |  | 0.211 |  |  | 0.369 |  |  | 0.404 |
| <30 years | 383 (76.3) | 119 (23.7) |  | 310 (61.8) | 192 (38.2) |  | 244 (48.6) | 258 (51.4) |  |
| ≥30 years | 438 (79.5) | 113 (20.5) |  | 355 (64.4) | 196 (35.6) |  | 282 (51.2) | 269 (48.8) |  |
| BMI, No. (%) |  |  | 0.108 |  |  | **0.029** |  |  | **0.005** |
| <24.0 kg/m^2^ | 655 (77.0) | 196 (23.0) |  | 524 (61.6) | 327 (38.4) |  | 407 (47.8) | 444 (52.2) |  |
| ≥24.0 Kg/m^2^ | 166 (82.2) | 36 (17.8) |  | 141 (69.8) | 61 (30.2) |  | 119 (58.9) | 83 (41.1) |  |
| **History of dysmenorrhea, No. (%)** |  |  | 0.052 |  |  | **0.008** |  |  | **0.002** |
| No | 657 (76.5) | 202 (23.5) |  | 522 (60.8) | 337 (39.2) |  | 404 (47.0) | 455 (53.0) |  |
| Yes | 148 (83.1) | 30 (16.9) |  | 127 (71.3) | 51 (28.7) |  | 106 (59.6) | 72 (40.4) |  |
| **Reproductive history** |  |  |  |  |  |  |  |  |  |
| Times of pregnancy, No. (%) |  |  | 0.249 |  |  | 0.255 |  |  | 0.287 |
| 0 | 362 (76.9) | 109 (23.1) |  | 302 (64.1) | 169 (35.9) |  | 236 (50.1) | 235 (49.9) |  |
| 1 | 377 (78.1) | 106 (21.9) |  | 306 (63.4) | 177 (36.6) |  | 249 (51.6) | 234 (48.4) |  |
| 2 | 73 (83.0) | 15 (17.0) |  | 52 (59.1) | 36 (40.9) |  | 38 (43.2) | 50 (56.8) |  |
| 3 | 9 (81.8) | 2 (18.2) |  | 5 (45.5) | 6 (54.5) |  | 3 (27.3) | 8 (72.7) |  |
| Times of delivery, No. (%) |  |  | 0.469 |  |  | 0.627 |  |  | 0.706 |
| 0 | 505 (77.3) | 148 (22.7) |  | 415 (63.6) | 238 (36.4) |  | 323 (49.5) | 330 (50.5) |  |
| 1 | 301 (78.8) | 81 (21.2) |  | 240 (62.8) | 142 (37.2) |  | 194 (50.8) | 188 (49.2) |  |
| 2 | 15 (83.3) | 3 (16.7) |  | 10 (55.6) | 8 (44.4) |  | 9 (50.0) | 9 (50.0) |  |
| **Infertility characteristics** |  |  |  |  |  |  |  |  |  |
| Type of infertility, No. (%) |  |  | 0.414 |  |  | 0.481 |  |  | 0.831 |
| Primary infertility | 368 (76.8) | 111 (23.2) |  | 308 (64.3) | 171 (35.7) |  | 241 (50.3) | 238 (49.7) |  |
| Secondary infertility | 453 (78.9) | 121 (21.1) |  | 357 (62.2) | 217 (37.8) |  | 285 (49.7) | 289 (50.3) |  |
| Etiology of infertility, No. (%) |  |  |  |  |  |  |  |  |  |
| Immune disease | 9 (90.0) | 1 (10.0) | 0.700 | 9 (90.0) | 1 (10.0) | 0.102 | 9 (90.0) | 1 (10.0) | **0.011** |
| Endocrine disease | 133 (87.5) | 19 (12.5) | **0.002** | 118 (77.6) | 34 (22.4) | **<0.001** | 95 (62.5) | 57 (37.5) | **0.001** |
| Uterine disease | 132 (88.0) | 18 (12.0) | **0.001** | 108 (72.0) | 42 (28.0) | **0.015** | 87 (58.0) | 63 (42.0) | 0.033 |
| Fallopian tube disease | 175 (76.4) | 54 (23.6) | 0.523 | 142 (62.0) | 87 (38.0) | 0.685 | 112 (48.9) | 117 (51.1) | 0.721 |
| Ovarian disease | 124 (87.3) | 18 (12.7) | **0.004** | 107 (75.4) | 35 (24.6) | **0.001** | 89 (62.7) | 53 (37.3) | **0.001** |
| Abnormity of sperm count or motility | 137 (94.5) | 8 (5.5) | **<0.001** | 119 (82.1) | 26 (17.9) | **<0.001** | 106 (73.1) | 39 (26.9) | **<0.001** |
| Unknown cause | 354 (71.1) | 144 (28.9) | **<0.001** | 269 (54.0) | 229 (46.0) | **<0.001** | 202 (40.6) | 296 (59.4) | **<0.001** |

BMI, body mass indexes; HSG, hysterosalpingography.
